# High-precision high-coverage functional inference from integrated data sources

**DOI:** 10.1186/1471-2105-9-119

**Published:** 2008-02-25

**Authors:** Bolan Linghu, Evan S Snitkin, Dustin T Holloway, Adam M Gustafson, Yu Xia, Charles DeLisi

**Affiliations:** 1 Bioinformatics Graduate Program, Boston University, Boston, MA, 02215, USA; 2 Center for Advanced Genomic Technology, Boston University, Boston, MA, 02215, USA

## Abstract

**Background:**

Information obtained from diverse data sources can be combined in a principled manner using various machine learning methods to increase the reliability and range of knowledge about protein function. The result is a weighted functional linkage network (FLN) in which linked neighbors share at least one function with high probability. Precision is, however, low. Aiming to provide precise functional annotation for as many proteins as possible, we explore and propose a two-step framework for functional annotation (1) construction of a high-coverage and reliable FLN via machine learning techniques (2) development of a decision rule for the constructed FLN to optimize functional annotation.

**Results:**

We first apply this framework to *Saccharomyces cerevisiae*. In the first step, we demonstrate that four commonly used machine learning methods, Linear SVM, Linear Discriminant Analysis, Naïve Bayes, and Neural Network, all combine heterogeneous data to produce reliable and high-coverage FLNs, in which the linkage weight more accurately estimates functional coupling of linked proteins than use individual data sources alone. In the second step, empirical tuning of an adjustable decision rule on the constructed FLN reveals that basing annotation on maximum edge weight results in the most precise annotation at high coverages. In particular at low coverage all rules evaluated perform comparably. At coverage above approximately 50%, however, they diverge rapidly. At full coverage, the maximum weight decision rule still has a precision of approximately 70%, whereas for other methods, precision ranges from a high of slightly more than 30%, down to 3%. In addition, a scoring scheme to estimate the precisions of individual predictions is also provided. Finally, tests of the robustness of the framework indicate that our framework can be successfully applied to less studied organisms.

**Conclusion:**

We provide a general two-step function-annotation framework, and show that high coverage, high precision annotations can be achieved by constructing a high-coverage and reliable FLN via data integration followed by applying a maximum weight decision rule.

## Background

Computationally based functional inference can be considered a two step process: (1) finding functional correlation between annotated and unannotated proteins by one or more experimental or computational methods, and (2) formulating a decision rule for transferring the function of annotated proteins to unannotated proteins. In general one can expect that the stronger the correlation, the greater the precision of transfer; similarly, one expects that the more data sources by which a correlation is found, the greater the precision. The integration of heterogeneous data sources by various methods is therefore an important component of the procedure [1-5].

In the first step, the result of a search for correlations between all proteins – annotated and unannotated – using any particular experimental or computational procedure, is conveniently displayed as a graph, the nodes representing proteins, and the links (edges) between them expressing a correlation. The links are generally weighted, reflecting the degree of correlation or functional similarity based on various experimental and computational evidence [1,3,6], such as physical interactions detected by yeast two hybrid experiments [7,8], correlated gene expression by microarray [9-11], and correlated phylogenetic profiles [12]. If the average number of links per protein is sufficiently large, the result will be a network of interactions, a so-called functional linkage network (FLN).

Although an FLN can be constructed by any of the above sources, different sources usually vary in reliability and coverage. For instance, while microarray and yeast two hybrid experiments can provide a great deal of information about the functional relationships between genes, they are noisy and subject to high false positive rates. While sophisticated statistical frameworks have been utilized to improve functional inference based on individual data sources [13-17], the coverage and reliability of single data source are inherently limited as one data type illuminates only limited aspects of the underlining biological mechanisms. To overcome these drawbacks, multiple data sources are often utilized and integrated using machine learning procedures, such as Bayesian methods, Neural Network, and Decision Tree. Previous integration results indeed support the intuitive expectation that such *integrated *FLNs can be more reliable than FLNs based only on a single data source [1,3,18-21].

**Figure 1:**
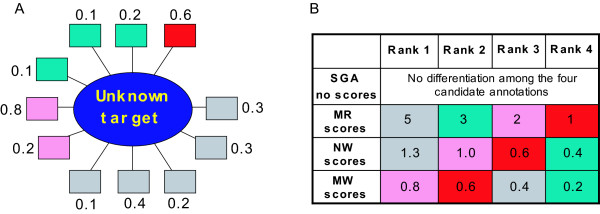
**Functional annotation decision rules**. (A): Local network representation of annotated neighboring proteins (rectangles) with weighted links to the unannotated target (circle). Color coding indicates the pathways in which the annotated proteins participate. Two metrics can be considered when determining annotation predictions of a target protein: (1) occurrence frequency of an annotation in the neighbors (2) weights of the relevant links contributing to a particular annotation. MR employs occurrence-frequency metric alone and weights a candidate annotation by counting neighbors having that annotation; e.g. for the grey annotation, the score is 5*1 = 5. NW emploits both metrics and uses a weighted sum of the links for the relevant annotation; e. g., for the grey annotation, the score is 0.1 + 0.4 + 0.2 + 0.3*2 = 1.3. MW employs linkage-weight metric alone and weights the annotations by the maximal linkage weight among all the linkages contributing to a particular annotation; for the grey annotation, the score is Maxf = 0.4. Table (B) shows the annotation ranking lists in descending order.

After FLN construction, the second step is network based functional inference [5,22-24]. Some methods have been developed which first identify network modules of related proteins [25-31] followed by annotation of these modules based on the known functions of its members. Alternatively, various decision rules can be employed to directly infer a protein's function based on its connections in the network. The simplest decision rule, standard guilty by association (SGA), casts the annotations of all neighbors to the unknown protein so long as the linkage weight exceeds a threshold [32-34]. Although SGA usually assigns at least one correct annotation to a target, the number of false positives is high due to lack of a selection metric to weight candidate annotations. Two factors could be considered as selection metrics to weight annotation predictions for a target protein: (1) occurrence frequency of an annotation among the neighbors (2) weights of the relevant links contributing to a particular annotation. To improve SGA, another decision rule, "majority rule" (MR), first applies SGA, and then weights the annotations in accordance with their occurrence frequencies [35]. It thus converts the annotations made by SGA into a rank ordered list. The performance of this method is dependent on where the list is truncated. Recently McDermott et al [36] suggested a generalization in which annotations are weighted by the sum of linkage weights. This "neighborhood weighting" (NW) procedure therefore considers both linkage frequency and weight. The above decision rules are illustrated in Fig 1. In addition to SGA, MR and NW which base annotation on local network connectivity, other algorithms are based on global network connectivity. Some methods repeat the application of MR or NW until the entire network reaches an extreme of some target function, for example (1) a minimum in the number of links connecting proteins with disparate functions, or (2) a maximum in the weighted sum of links connecting proteins sharing the same functions [6,37,38]. Another framework exploiting global network connectivity applies a Markov random field (MRF) model by calculating the probability that a protein has a function given the functions of all other proteins in the network [39,40]. Additionally, a flow based method has also been developed, which simulates the functional annotation of proteins as the spreading of functional flow in a FLN [41].

In a weighted FLN, network based functional inference can achieve high precision when using a stringent network linkage weight cut-off, but that comes at the price of low proteome coverage. Alternatively, when a less stringent linkage weight cut-off is used, frequency-based inference rules can achieve good coverage, but at the expense of a high false positive rate. To address this challenge, using *Saccharomyces cerevisiae *as a model organism, we explore a two-step function-annotation framework: (1) construction of a high-coverage and reliable FLN through the integration of heterogeneous data sources using machine learning methods; (2) development of a matching decision rule for the constructed FLN to optimize functional annotation. The goal of this study is to provide precise functional annotation for as many proteins as possible by combination of both steps. We choose KEGG [42,43] as our functional ontology because its endpoint, pathway presence, is relatively well defined. GO is also useful and has been employed extensively [1,34,36,44-46], but the inherent variance in GO tree depth for different functional families can complicates test statistics. In the first step, we find that various machine learning methods can all combine diverse sources to construct a high-coverage and relatively reliable FLN with comparable quality. In the second step, we introduce an adjustable decision rule. Empirical tuning of this adjustable rule for the constructed FLN results in a relatively simple *maximum weight *(MW) rule, which uses maximum linkage weight to assign functional annotation. We find that MW increases the precision over other methods, and at high coverage it can be up to 2.5 fold more precise than MR and NW, and up to a 25 fold more precise than SGA. We also develop a scoring scheme to estimate annotation precision for individual predictions.

## Results and discussion

### General procedure

**Figure 2:**
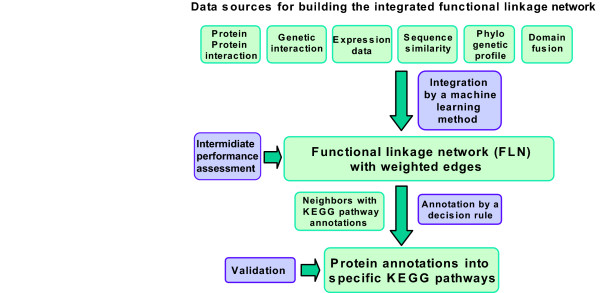
**Overview of methodology**. The framework can be divided into two steps: (1) construction of an integrated functional linkage network (FLN) (2) development of a decision rule for the constructed FLN to optimize functional annotation. Green boxes denote data inputs or products; purple boxes denote actions. In step one six data sources are used as inputs to one or another learning algorithm, to find functionally associated pairs of proteins. The linked proteins identified thus comprise a weighted functional linkage network. In step two, proteins of unknown functions are then annotated based on the collective properties of neighboring annotated proteins, using one or another decision rule. Performance, which we measure by a combination of precision and coverage, is evaluated as described below, using doubly annotated protein pairs.

The overall procedure (Fig 2) consists of two steps. The first step combines various data sources by a machine learning method to construct a high sensitivity network such that for any node (protein), at least some nearest neighbors are likely to be functionally related. In the second step, various decision rules are then explored for the ability to prune the set of functional linkages to obtain high precision annotation while still covering a large number of proteins.

### FLN construction by machine learning classifiers

**Figure 3:**
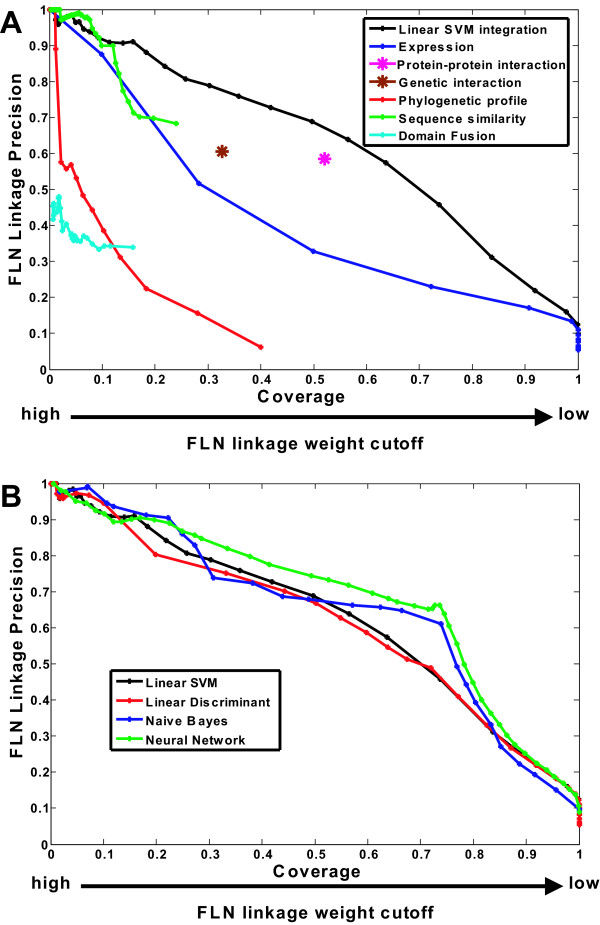
**Linkage precision against coverage for FLNs**. (A): Integrated FLN performance substantially exceeds those obtained using single data sources. The FLN linkage precision-coverage curves are plotted by varying the FLN linkage weight cutoffs as described in method section. PPI and GI networks have equal-unit weighted linkages and thus have only one data point denoting one coverage and one precision. (B): FLNs integrated by the four classifiers perform comparably.

A machine learning classifier is applied to assign linkage weights, a measure of tendency of pathway sharing, to protein pairs by incorporating information from multiple data sources as described in Methods section. The result is a functional linkage network (FLN). A high-quality FLN would have both high linkage precision and high proteome coverage [3], where the number of "covered" proteins is the maximum number of proteins that can be annotated. We expect that a principled integration of multiple information sources will result in performance that is better than for any single source. A comparison of performance between integrated sources obtained using a linear SVM (support vector machine with a linear kernel) and single sources, clearly confirms this expectation (Fig 3A). In addition to linear SVMs, we use Linear Discriminant Analysis, Naïve Bayes, and Neural Network, and find that all four have comparable integration performance (Fig 3B).

### Decision rules for prediction of protein function

Given a network of linked proteins and correlations assigned to each link based on integrated data sources, the problem is to assign unannotated proteins to KEGG pathways, with a high precision. To this end we propose an adjustable decision rule, with a single parameter alpha. The rule is such that the score relating a protein to a particular annotation is the sum of edge weights which link the unannotated protein to proteins with the given annotation, raised to the power alpha (equation 3, see Methods). To determine the optimal alpha value for application to the *S. cerevisiae *FLN we select three alpha values which correspond to special instances of the decision rule. First, we select an alpha value of zero, which is equivalent to the previously described majority rule decision rule (MR) [35]. With the MR rule, annotation frequency among neighboring proteins is used to determine the annotation score. Second, we select an alpha value of one, which is equivalent to the previously described neighborhood weighting rule (NW) [36]. With NW, both the edge weights and frequency of annotation are considered, as the sum of the edge weights of neighbors belonging to a particular annotation category determine the score for that category. Finally, we test a maximum weight rule (MW), which is the rule approached as alpha becomes infinitely large. With the MW rule, an annotation score for a particular annotation category is determined by the maximum edge weight among neighbors belonging to that category. In addition to the MR, NW, and MW rules derived from our adjustable decision rule, we also evaluate the naïve standard guilt-by-association (SGA) [32-34], where all annotations of neighbors are assigned without considering edge weights or frequency.

#### Rank and linkage weight cut-off affect annotation performance

**Figure 4:**
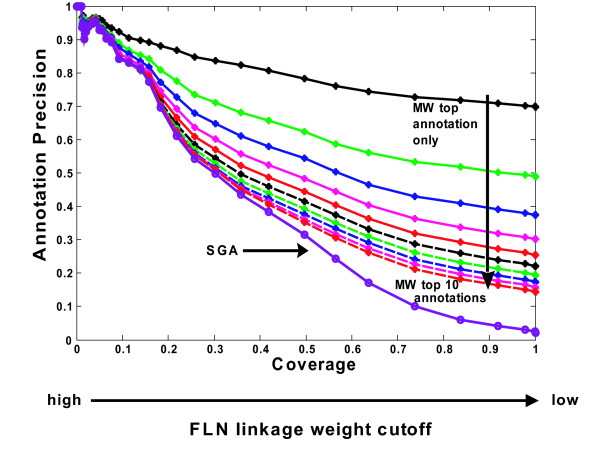
**Rank and linkage weight cutoff affect annotation precision in MW**. The x axis (fraction of proteins having predicted annotations) is obtained by varying the FLN linkage weight cutoff. The family is generated by varying annotation rank cutoff from 1 (upper most curve for which only the top ranked annotation is included) to 10.

The performances of MR and NW were previously indicated to be determined by two parameters, linkage weight cut-off and rank cut off (i.e. for the same target protein all predicted annotations exceeding a prespecified rank are assigned) [36]. These two factors also determine the performance of annotation precision-coverage characteristic curve (APCC) obtained using MW (Fig 4). In particular, for each linkage weight cut-off, the candidate annotations of all neighbors of a protein are listed in descending order according to equation 2. A cut-off of 1 means only the highest ranked annotation is used; a cut-off of 2 means the top 2 annotations are assigned etc. Schwikowski, et al. applied a similar procedure for selecting predictions with a fixed rank cut-off [35]. The result clearly shows that the best APCC is obtained by using the most stringent rank cut-off; i.e. the top ranked annotation. As the rank cut-off drops, MW loses precision, with an increasing number of false positives, and in the limit gives the same APCC as SGA.

#### Comparative performances of decision rules

**Figure 5:**
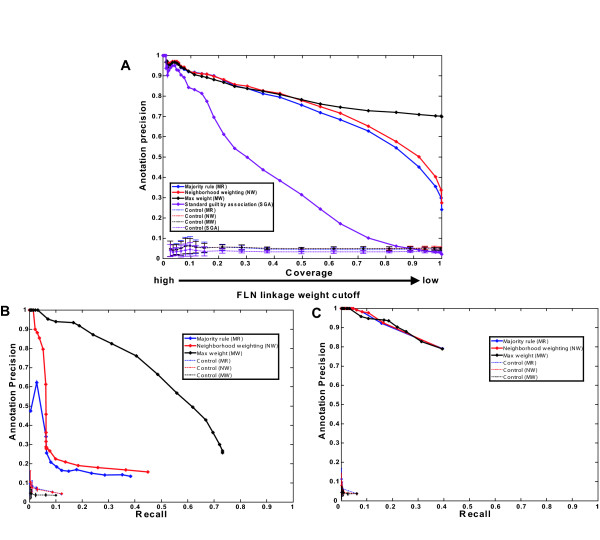
**Comparison of decision rules**. (A): Comparative performance of MR, NW, MW, and SGA in annotation precision-coverage analysis. Six data sets are integrated using a linear SVM to construct a weighted FLN. Given a prespecified linkage weight cutoff, MR, NW, and MW use the highest ranked predicted annotations for comparison, SGA takes all the candidate annotations as predictions without ranking. A coverage of 1 means all 5475 proteins can be assigned at least one function. (B): Comparative performance of MR, NW, and MW in annotation precision-recall analysis in a high-coverage and relatively noisy FLN composed of 5475 proteins. (C): Comparative performance of MR, NW, and MW in annotation precision-recall analysis at a low-coverage and relatively confident FLN composed of 1872 proteins. In all the figures, the same evaluations are repeated in random control networks as dashed lines with error bars denoting one standard deviation of 10 repeating runs.

Linkage weight cut-off and rank are common factors affecting precision in MR, NW, and MW, though each has its own criterion of assigning rank, and the best precision at a given coverage is obtained by assigning only the highest ranked annotation. A comparison of the annotation precision-coverage curves for the different decision rules using only the top ranked annotation indicates that (1) as expected, SGA is worse than the other three at all degrees of coverage and (2) the other three rules perform comparably at low coverage (less than 0.35), but MW diverges rapidly from the other two for coverage greater than 0.6, remaining precise at all degrees of coverage (Fig. 5A). Comparisons with the top 2 or 3 ranked predictions show similar trends. These results are obtained using linear SVM, but similar results are obtained using other classifiers (Fig. S1) [see Additional file 1]. In addition, we also compare MR, NW, and MW rules by annotation precision-recall analysis and obtain similar results. At a low linkage-weight cut-off (a high-coverage and relatively noisy network), MW outperforms NW and MR (Fig. 5B); At a high linkage-weight cut-off (low-coverage), the three decision rules have similar performance, all with low recall (less than 0.4) and high precision (over 0.8) (Fig. 5C). The performances of these decision rules are also evaluated in random control networks. The results indicate that relative to performances with the real network, the random control curves for different annotation methods have roughly similar poor performances (Fig. 5). Annotation precision-recall analysis using yeast Gene Ontology slim terms (downloaded from Saccharomyces Genome Database) as the annotation ontology shows similar trends (Fig. S3) [see Additional file 1].

The superior performance of MW can be understood by considering the properties of the integrated FLN and the effect of decreasing the linkage cut-off in an attempt to increase proteome coverage. In the FLN derived from integration of multiple data sources with machine learning, the linkage weight is proportional to linkage precision (assessment of tendency of pathway sharing of linked proteins) (Fig. 3A). Therefore a low linkage weight cut-off generates a high-coverage and relatively noisy network, and a high linkage weight cut-off generates a low-coverage and relatively confident network. As the linkage weight cut-off is decreased, and more low confidence links are incorporated in the network, MR and NW by definition consider all these weak links in determining the ranks of different annotations (equation 1). Furthermore, MR weights strong and weak links equally and thus performs worse than NW. MW on the other hand, selectively uses only the highest quality link contributing to each relevant annotation, to rank different annotations (equation 2 and Fig. 1) and therefore acts as a better noise filter on a high-coverage and relatively noisy FLN.

**Figure 6:**
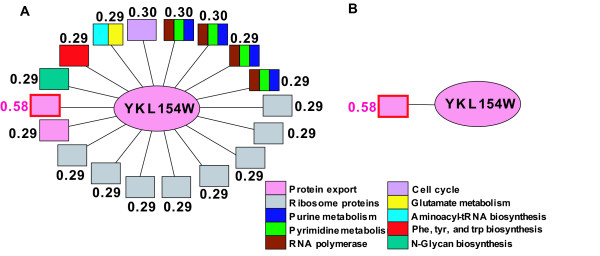
**Local network representation of annotated neighboring proteins (rectangles) linked to the unannotated YKL154W (circle)**. Color coding indicates the pathways in which the annotated proteins participate. (A): A linkage weight cutoff of around 0.29 generates a dense and noisy FLN. (B): A linkage weight cutoff of 0.58 generates a sparse and accurate network.

To fix ideas, consider the unannotated yeast protein YKL154W, which is linked to 18 annotated neighbors participating in 10 pathways (Fig. 6A). SGA, by definition, chooses the annotations of all neighbors. The other 3 methods prune the results in accordance with rank cut-off. At a weight cut-off of 0.58 (a sparse and accurate network, Fig. 6B), all methods choose "protein export" as the only prediction. However, when the much less stringent linkage weight cut-off of 0.29 is used (a dense and noisy network), the top ranked prediction for MW is "protein export", while NW and MR choose "ribosome proteins" due to its high occurrence frequency. Although YKL154W is not annotated in KEGG, it is annotated in SGD, described as "Signal recognition particle (SRP) receptor beta subunit; involved in SRP-dependent protein targeting; anchors Srp101p to the ER membrane". In KEGG database, SRP (signal recognition particle) dependent protein targeting process is defined to belong to "protein export" pathway. SRP receptor beta subunit is not part of the ribosome complex, thus "protein export" is the most appropriate prediction. Only MW ranks "protein export" as the top prediction independent of the network quality.

**Figure 7:**
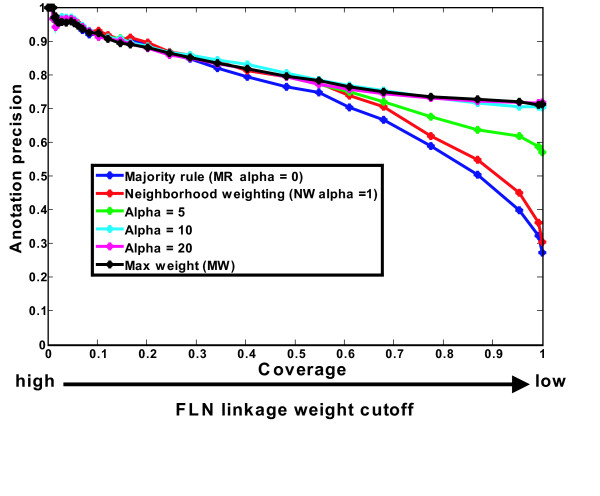
**MW decision rule is the end result of carefully tuning an adjustable decision rule**. The adjustable decision rule (equation 3) is tuned to optimize function-annotation performance for the integrated FLN by empirically testing a range of alpha values. MR, NW, and MW rules are special cases derived from this adjustable rule, with alpha set to be 0, 1, and infinity, respectively. When alpha is equal or above 10, the optimal performance is obtained, which is approximates to the performance of MW. The performances are evaluated by the annotation precision-coverage curves.

Above we have compared MW with MR and NW, which are special instances of our adjustable decision rule, with specific values of the alpha parameter. To make sure that there is not some intermediate alpha value which is in fact optimal, we empirically test a range of alpha values and as shown in Fig. 7 and the result shows that performance increases with alpha, indicating that MW is the optimal rule.

**Figure 8:**
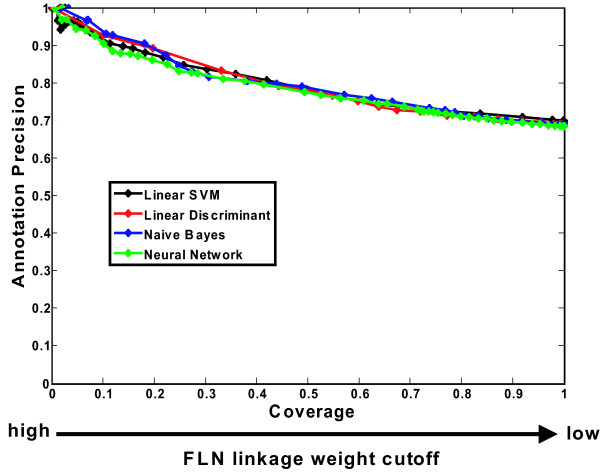
**Performances of MW using different machine learning methods to construct functional linkage networks (FLNs) are comparable**. Performance of MW using each of the machine learning methods to construct a weighted functional linkage network (FLN) as input is evaluated in annotation precision-coverage analysis.

An obvious question to consider is how performance of the MW decision rule varies with choice of classifiers. As is evident from Fig. 8, the annotation precision-coverage curves generated using MW with different classifiers are virtually super imposable. So at least for the 4 classifiers considered in this paper, which one is used is more or less irrelevant – all that matters is the decision rule.

### Generation of final predictions and estimate prediction precisions using MW scores

Although the four classifiers show similar performance in combination with MW decision rule, we have some preference for using a linear SVM classifier because (1) it is linear and therefore simpler (linearity rules out neural nets) and (2) Naïve Bayes and Linear Discriminant Analysis require additional assumptions about input data [see Additional file 1].

**Figure 9:**
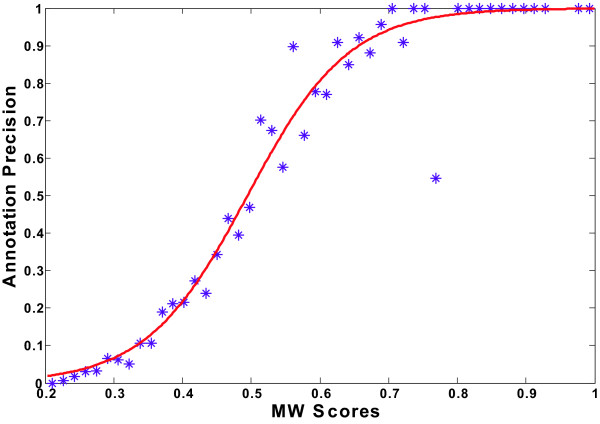
**Calibration curve for estimating annotation precisions from MW scores**. The x axis denotes MW scores; the y axis denotes annotation precision. The predictions are binned into 50 equally spaced intervals based on associated MW scores. Annotation precision for each bin is calculated based on the comparisons of the predicted annotations and the original annotations for known proteins. The purple dots denote the binning results. The red curve shows curve fitting results with a two parameter logistic regression function, y = exp(a + b*x)/(1+ exp(a + b*x)). a = -6.71 and b = 13.57 (with 95% confidence bounds).

Although the best overall precision is obtained by assigning the top ranked annotation only, and full coverage can be achieved with high precision, proteins have multiple functions, and many of these will be missed. A more informative procedure would be to choose the top few annotations, evaluate the precision of each, and then pick those annotations that exceed a prespecified precision. In particular, we begin with a low weight cut-off of 0.2 to obtain a full-coverage FLN, and then choose an arbitrary rank cut-off, for example top 5, to assign up to 5 annotations per protein, with each annotation associated with a MW score. Finally we estimate a specific annotation precision for each prediction by calibrating the MW scores against annotation precision [18] (Fig. 9).

The calibration curve is obtained by binning the MW scores, assigning a precision estimate to each bin by comparing the predicted annotations and the original annotations for known proteins (equation 4a), and then fitting the results to a logistic regression function. The result is shown in Fig 9, using 50 equal bins. The good match between the binning results and the fitted curve suggests that the MW score is positively correlated with annotation precision and can be used to estimate the latter. For instance, for YKL154W (Fig. 6A), only the most appropriate prediction, "protein export", has a high estimated precision of above 0.7, all other predictions are less than 0.1. More examples are also provided [see Additional file 2]. We list pathway predictions for all the proteins covered in input data sources, each associated with a precision estimate [see Additional file 3].

We summarize our prediction results by filtering the predictions with increased MW score cutoffs and evaluating the predictions based on proteins with known annotations (Table 1). For instance, at precision of 0.6 and recall of 0.53 (calculated based on equation 4a and equation 4b), with 1161 proteins among 99 pathways as annotation source, 638 unannotated proteins are assigned into 1499 specific pathways. We are able to assign multiple pathways to a protein: from precision of 0.4 to 0.9, up to 5 pathways can be assigned to a protein and the average number of pathways per protein is larger than 1.4. Our predictions cover large number of pathways. Even at high precision of 0.8, our method still enriches 59 pathways, and as the KEGG database continues to expand, we expect methods such as those developed here to provide increasingly comprehensive understanding of protein function and pleiotropy.

**Table 1:** Prediction result summary

MW score cutoff	Annotation Precision	Recall	Number of KEGG unannotated proteins covered	Number of predictions for KEGG unannotaed proteins	Average predicted pathways per protein	Unique pathways in predictions
0.33	0.4	0.68	2176	6200	2.85	97
0.38	0.5	0.61	1218	3176	2.61	93
0.43	0.6	0.53	638	1499	2.35	81
0.47	0.7	0.46	347	739	2.13	64
0.50	0.8	0.39	242	493	2.04	59
0.58	0.9	0.22	87	173	1.99	36

### Evaluation of framework robustness and potential for applicability to less well studied organisms

We have demonstrated that our framework performs well in yeast. Similarly, we have applied linear SVM in combination with MW rule in *E. coli *and we find that the framework also performs well (Fig. S2) [see Additional file 1]. While these results are encouraging, we are aware that our MW rule is a linkage weight based local decision rule, i.e. uses only immediate network neighbors for functional prediction. As a local decision rule, MW will tend to perform best with a network where on average unannotated proteins are well connected to annotated proteins with high precision links. For an integrated FLN, these network properties are primarily dependant on the number of annotated proteins in the network and the availability of diverse data sources. With our analysis in yeast and *E. coli*, our FLNs meet these ideal criteria, with the level of annotation being relatively high, and multiple high quality data sources being integrated using machine learning.

However, less studied organisms likely won't have as many annotated proteins or data sources available. In order to more rigorously assess whether our framework is likely to meet with success when applied to less studied organisms, we have performed analyses in yeast and *E. coli *to test how the performance is affected by (1) decreasing the number of annotated proteins in the network and (2) reducing data sources to include only those which are likely to be available for most organisms [see Additional file 1]. We find that using just 500 annotated proteins (less that half of the total available annotated KEGG proteins) as annotation source, the precision at various degrees of coverage is still over 0.58 in yeast and over 0.48 in *E. coli *(Fig. S5a and S5b) [see Additional file 1]. Additionally in yeast, we leave out high-throughput PPI (protein-protein physical interaction) and GI (genetic interaction) data as those data are not available for most eukaryotes. Using only sequence based phylogenetical profile, domain fusion, and sequence similarity as well as expression data, the precision remains over 0.6 at various degrees of coverage in yeast (Fig. S5c) [see Additional file 1]. In *E. coli*, using only sequence based data sets (e.g. phylogenetic profiling, fusion, chromosomal proximity, gene cluster, and sequence similarity), as these data sets will be readily available for any sequenced prokaryote, the precision remains over 0.7 at various degrees of coverage, although the coverage does drop to 0.75 (Fig. S5d) [see Additional file 1]. In both tests the performance reduction is small relative to the overall performance for both species, and it is therefore likely that our framework can be applied to less studied organisms.

Although these analyses demonstrate some degree of robustness in our framework, in a very poorly annotated organism with few available data sources, most unannotated nodes will on average have few annotated neighbors, resulting in relatively little additional annotation. In such a situation methods that use new (inferred) annotations as they are assigned, could increase performance [6,37,40,41], The reliability of the final annotated network will, however, be critically dependent on the quality of local inferences; i.e. the local decision rule remains crucial. The approach we have taken here, which uses KEGG annotations, starts with a relatively sparsely annotated network, since only around 20% of *Saccharomyces cerevisiae *genes are KEGG annotated. Many genomes of interest exceed this annotation percentage, so even in its present form of being strictly local, we can expect a fair degree of general applicability, despite the fact that there may indeed be FLNs in which a global decision rule is preferable.

To demonstrate the importance of the local decision rule, we have compared the annotation performance of the MW rule with the previously published functional flow method [41], using the integrated yeast FLN as the test network. The functional flow method exemplifies global decision schemes, as node annotation includes the iterative application of a local rule as a means to propagate annotation from non-adjacent nodes. As a first step in our analysis, we compare MW to functional flow, with propagation limited to the adjacent node (Fig. S4 (d = 1)) [see Additional file 1]. This is in effect a comparison of MW to the local decision rule used by functional flow, as there is no propagation of information beyond neighboring nodes. As the local decision rule underlying the functional flow algorithm is a variant of the NW rule, we find that unsurprisingly, MW shows performance increases similar to those seen in the previous comparison to standard NW at high coverage. Additional comparisons in which functional flow is implemented with varying propagation distances show similar results, indicating that the benefit of global propagation is unable to compensate for the use of a suboptimal local decision rule. Despite this comparison, we expect that a method which incorporates the optimal local decision rule into a global framework will result in further improvement in results, and will be a subject of future work.

### Additional considerations for future application of the proposed framework

In addition to potential performance improvements achieved by embedding our local decision rule in a global framework, we also expect that small improvements can be gained in the future with more sophisticated data handling. Specifically, some data sources such as PPI and gene expression are noisy, which has the potential to result in spurious linkage weights. Although our machine learning based data integration substantially improves the reliability and coverage of linkages when compared to linkages based on individual data sources (Fig. 3), it is possible that if we adopted some of the more sophisticated methods used to remove noise and improve functional inference with microarray and PPI measurements [13-17], we might further improve performance. Our currently analysis and conclusion about MW are based on experiments in yeast and *E. coli*. In the future, we plan to further explore the application and extension of the framework in other species, especially higher eukaryotes such as human.

## Conclusion

In this study, we have developed a general two-step function-annotation framework, and show that high coverage, high precision annotations can be achieved by constructing a high-coverage and reliable FLN through integrating multiple data sources using a machine learning method, followed by the application of an adjustable decision rule. For FLN construction, we demonstrate four commonly used machine learning techniques all show comparable performance. For decision rule selection, MW rule is identified as optimal for the integrated FLN after the tuning of the adjustable decision rule. Taking advantage of the correlation between MW score and prediction precision, a scoring scheme to estimate the precisions of individual predictions is also provided. Finally, we also test the robustness of our framework and find that our framework can likely be applied to less studied organisms.

## Methods

### Machine learning classifiers to construct FLN

We use supervised procedures that learn to recognize whether protein pairs are in the same pathway based on six features: sequence similarity, protein domain fusion, correlated phylogenetic profiles, expression profiles, physical interactions and genetic interactions. For the purpose of exploring network-based functional annotations after FLN construction, similar as Barutcuoglu, et al.[47], we use unthresholded outputs for a machine learning classifier, i.e. all the outputs are unbounded real values instead of binary labels. Specifically, the output is a vector each of whose elements weights the tendency of pathway sharing of a pair of proteins in the input set, with the weights normalized between 0 and 1. The details for linkage weight calculation are described using linear SVM as an example [see Additional file 1].

We compare the performance of four common machine learning classifiers for building FLNs including linear kernel Support Vector Machine (linear SVM), Linear Discriminant Analysis, Naïve Bayes, and a feed-forward Neural Network with one hidden layer [see Additional file 1]. We train our soft maximum margin SVM using the SPIDER package, with a wide range of penalty functions for misclassification [48]. All other classifiers are from PRTools package implemented with default parameters.

#### Gold standard (GS)

The input set includes only protein pairs for which both members have RefSeq protein sequences and microarray expression measurement since the sequence data and expression data have the largest proteome coverage. This ensures the integration by providing at least four input features, three sequence based features and one expression correlation feature. The final set consists of 5475 proteins, or nearly 15 million pairs. Of these, 26,920 co-occur in at least one KEGG pathway, and we take them as our gold standard for true positives (GSP). We define our true negative gold standard (GSN) as the collection of pairs that: (1) are annotated in KEGG; (2) never occur in the same KEGG pathway based on current knowledge; (3) are found experimentally to be in different subcellular locations. There are 234,141 such pairs. Franke et al used similar criteria for selecting GSN [49].

#### Data sources for encoding input features

Each of the six sources, sequence similarity, protein domain fusion, phylogenetic profile, microarray expression, protein-protein interaction, genetic interaction, contributes one component to the feature vector characterizing a protein pair.

##### (1) Sequence similarity

We download protein sequences from RefSeq [50,51] and use blastp in blast2.2.13 to perform an all against all blast within the proteome. Pairs are filtered by requiring that their best alignment has an E-value exceeding 0.1 and the smaller protein aligns to the larger in at least 50% of its length. A total 10,868 pairs involving 3264 proteins pass this filter, and their E-value serves as the input feature. Pairs not passing the filter take the default E-value of 1.0.

##### (2) Protein domain fusion

Some protein pairs with similar functions fuse into different domains of one single protein in other species [52]. We implement the domain fusion method as described in the Prolink database and calculated a P-value denoting the probability of a fusion event occurring by chance as the input feature [53]. Total 9618 fusion pairs among 1554 proteins were generated. Pairs not having fusion events take the default P-value of 1.0.

##### (3) Phylogenetic profile

The presence and absence of a protein across a set of genomes can be represented by a binary string, its phylogenetic profile. Proteins with sufficiently similar profiles tend to be functionally related [12,54]. As a measure of correlation, mutual information is used as the input feature [54]. Pairs not having correlated phylogenetic profiles take the default value of 0. Total 2,384,295 pairs have non zero mutual information in 2187 proteins.

##### (4) Microarray expression

Expression data are from the three major yeast datasets [9-11], composed of 6206 proteins in 551 experimental conditions. These gene expression datasets consist of log transformed expression level ratios. We choose genes included in all the three datasets and combined the experimental conditions. The final data set is composed of 6206 genes in 551 experimental conditions. The missing values were estimated by KNN imputation [55]. Next we normalized the data such that the mean of measurements from each array is 0 and standard deviation is 1. We compute the Pearson correlation coefficient for every pair as the input feature for integration [2,5,30].

##### (5) Protein-protein interactions (PPI)

Tyers et al. recently published two large yeast interaction data sets, one for protein-protein interactions and other for genetic interaction [56]. We use the union of high-throughput PPI and literature curated PPI as inputs. All the PPI subtypes (Two-hybrid, Affinity Capture-MS, Affinity Capture-Western, and Co-purification) are included, except for co-localization, which is used to establish a true negative gold standard. In total 20,121 pairs among 4659 proteins are included with self interaction and redundant interactions removed. A binary value serves as the input feature denoting existence or absence of an interaction.

##### (6) Genetic interactions (GI)

All the subtypes of genetic interaction data including synthetic lethality, synthetic growth defect, dosage lethality, dosage growth defect are included, for a total of 13,340 pairs among 3052 proteins [56]. We also use a binary value as the input feature.

#### Training/classification/validation procedures

For each of the four machine learning methods we train 30 individual classifiers, followed by aggregation for final predictions.

##### (1) Training/classification of individual classifiers and classifier aggregation

Typically a classifier can be constructed simply by training a learning algorithm on the entire set of GS proteins. A cross validated functional linkage network is then built by applying the trained algorithm to all other available protein pairs. This strategy is appropriate when the final result is only the FLN. There are several considerations which prompt us to use a more sophisticated design. Firstly, performance characteristics for the FLN and validation of the annotation decision rule are desired. To provide a fair evaluation of the annotation step, all of the links in the FLN should be based on *out-of-sample pairs*. i. e., weighted links between proteins will be retained in the FLN only when those proteins are not in the training set. Our method addresses this issue by repeatedly splitting the gold GS pairs into individual training segments to train individual classifiers, and aggregating the predictions of those individual classifiers on the left-out pairs as final predictions. Specifically, for each protein pair in the input set, the final prediction is determined by taking the median value of the predictions of only those individual classifiers for which neither member of the pair is included in the relevant training set. Barutcuoglu et al. [47] used a similar approach. Details are also provided [see Additional file 1].

##### (2) Performance characteristics of the FLN

For each machine learning method, the final output after classifier aggregation is a vector each of whose elements weights the tendency of pathway sharing of a protein pair in the input set, for both non-GS and GS pairs, the latter being used to assess performance characteristics. Specifically, similar to Lee et al., [3] we evaluate linkage precision against coverage by varying the linkage weight cut-off to differentiate positive from negative predictions.

*FLN linkage precision*: given a prespecified linkage weight cut-off, precision is the fraction of all positive predictions that are correct; i.e. the pairs sharing at least one pathway. This precision is estimated by comparing the predictions of GS pairs and their original class labels.

*Coverage*: since we use annotated neighbors to assign functions to unknown proteins, coverage is defined as the fraction of proteins linked to at least one annotated neighbor, i.e. the fraction of proteins for which a functional assignment can be made.

### Network-based protein function predictions

The following network-based decision rules assign functions to unannotated proteins in a FLN, using the concept of guilt by association, i.e. proteins tend to have functions similar to those of their interacting partners. As annotation source, we use KEGG pathways, which include 1161 proteins among 99 specific pathways in our FLN.

#### Decision rules (Fig 1)

##### (1) Standard Guilt by association (SGA)

SGA assigns all annotations of each neighbor whose link to the target protein exceeds a prespecified cutoff without differentiation [32-34].

##### (2) Neighborhood weighting (NW) [36]

Let *w*_*i *_be the weight of the ith neighbor that has annotation *v*. Then the score for annotation *v *is

(1) $$S_{v}=\sum_{i=1}^{N}w_{i}$$

where N is the number of neighboring proteins having annotation *v*. NW weights the annotations returned by SGA by *S*_*v*_.

##### (3) Majority Rule (MR)

SGA outputs are weighted by *S*_*v *_in equation 1 with all *w*_*i *_set to 1[35].

##### (4) Maximum weight (MW)

Candidate annotations are assigned in accordance with the maximal weight of the links contributing to a specified annotation.

(2) *S*_*v *_= *Max*{*w*_*i*_} *i *= 1,2, ..., *N*

##### (5) Adjustable decision rule

The adjustable decision defines the annotation score as equation 3.

(3) $$\begin{array}{cc} S_{v}=\sum_{i=1}^{N}w_{i}^{\alpha } & 0<w_{i}<1,\alpha \geq 0 \end{array}$$

*α *is an ajustable integer parameter to be tuned to optimze performance for a particular input FLN. As described in background section, two factors can be considered regarding selection metrics when annotating a target protein: (1) occurrence frequency of an annotation in the neighbors (2) weights of the relevant links contributing to a particular annotation. Continuously increasing *α *from 0 allows to flexibly adjusting the contributions of the two such that lower weighted links have smaller impact on dermining the final annotation score. Consiquently a broad spectrum of decsion rules are generated, ranging from soley frequency based MR (*α *= 0) rule at one end to solely linkage weight based MW rule (when *α *is infinitly large) at the other end. NW is a special case (*α *= 1) emploiting both factors in the middle.

#### Assessment of annotation performance

The annotation performance of a decision rule is evaluated in a leave-one-out setting using the annotated proteins, i.e. every protein with known annotations is held out as the target protein for function prediction based on the annotations of all other proteins by a decision rule [57]. The *annotation precision *and *sensitivity (or recall) *are estimated by comparing the original annotations and the predicted annotations. In particular [57], for protein *i*, let *n*_*i *_be the number of known annotations; *m*_*i *_the number of predicted annotations, and *k*_*i *_the overlap count between the predicted and true annotations. Then

(4a) $$Annotation\;precision=\sum_{i}k_{i}/\sum_{i}m_{i}$$

(4b) $$Sensitivity(recall)=\sum_{i}k_{i}/\sum_{i}n_{i}$$

We also use *Coverage*, the fraction of the set of 5475 proteins (total number of yeast proteins covered in the six input data sources) having at least one prediction in the FLN at a given linkage weight cutoff as another assessment, i.e. proteins linked to at least one annotated neighbor in the FLN. An overall coverage of 1 indicates that all of the 5475 proteins are linked to at least one annotated protein and thus have at least one predicted annotation. Annotation coverage increases as the linkage weight cutoff becomes less stringent because more links are being included in the network.

Since our goal is to assign precise annotations to as many as possible proteins. We use *annotation precision*-*coverage *curve for comparing different decision rules as described below [3,24,36,54]. First, using data integration techniques, we calculate the functional linkage weights for all protein pairs covered by the six input data sets (5475 proteins in total). These links are then filtered by different linkage weight cut-offs, which has the effect of generating functional linkage networks (FLN) with different proteome coverages. At each linkage weight cutoff a given annotation method is applied to generate a ranked list of putative annotations for each protein and top 1 ranked annotations are used to evaluate different annotation methods. The reason we evaluated the methods using only the top 1 ranked annotation for each protein is because the top ranked predictions have the highest annotation precision (Figure 4) and thus we are comparing the best predictions made by each method. Since we have only one prediction for each protein after selecting top 1 ranked predictions, based on the definition of Equation 4a, the annotation precision at a given cut-off is then determined by calculating the fraction of KEGG proteins whose top ranked prediction matched their actual annotation. Coverage is calculated as described above for each tested linkage weight cut-off.

## Authors' contributions

BL designs and implements the whole computation frame work. DH designs and implements all the machine learning classifiers. ES and AG compute part of the data sources for integration and design decision rules. YX monitors the whole framework. CD designs and directs the whole project and is Principal Investigator on the NIH grant that funded the project. All the authors have read and agreed to the manuscript.

## Supplementary Material

Additional File 1 Supplemental material. Supplemental material provides additional detail descriptions about the following. (1) Brief descriptions of the four machine learning classifiers. (2) training/classification of individual classifiers and classifier aggregation. (3) Compare decision rules based on FLNs constructed by the other classifiers in yeast. (4) Detailed descriptions about two more prediction examples. (5) Compare the four decision rules for functional annotation in *E. coli *using linear SVM integrated FLN. (6) Compare decision rules in annotation precision-recall analysis. (7) Compare MW decision rule and functional flow algorithm. (8) Test robustness of the framework by reducing data sources or annotation sources. (9) Perform random control experiments for each evaluated annotation method. Click here for file

Additional File 2 Prediction examples. Predicted annotations of un-annotated proteins not covered by KEGG pathways are defined as novel predictions, though some might have annotations in other databases such as SGD or MIPs [58,59]. We list more examples to show that our novel predictions with high estimated precisions represent appropriate pathway assignments using annotations in MIPs as supporting references. ORF names of proteins, predicted KEGG pathways by MW decision rules, estimated precisions based on the curve fitting function in figure 9, and annotations from MIPs database as supporting references are listed. Click here for file

Additional File 3 Function-annotation predictions in yeast. List of predictions. Column 1: ORF name; column 2: predicted pathway annotation in KEGG pathway ID; column 3: MW score; column 4: estimated annotation precision; total number of proteins: 5475; total number of predicted annotations: 27,319; up to 5 annotations are predicted for each protein. Click here for file
