# Supplementary material for: High-precision high-coverage functional inference from integrated data sources
Source: BMC Bioinformatics. 2008 Feb 25;9:119. doi: 10.1186/1471-2105-9-119 (PMC2292694; doi:10.1186/1471-2105-9-119)
Supplement: Additional File 1 — Supplemental material. Supplemental material provides additional detail descriptions about the following. (1) Brief descriptions of the four machine learning classifiers. (2) training/classification of individual classifiers and classifier aggregation. (3) Compare decision rules based on FLNs constructed by the other classifiers in yeast. (4) Detailed descriptions about two more prediction examples. (5) Compare the four decision rules for functional annotation in E. coli using linear SVM integrated FLN. (6) Compare decision rules in annotation precision-recall analysis. (7) Compare MW decision rule and functional flow algorithm. (8) Test robustness of the framework by reducing data sources or annotation sources. (9) Perform random control experiments for each evaluated annotation method. [file 1471-2105-9-119-S1.pdf]

Supplemental material

1. Brief descriptions of the four machine learning classifiers [1]

1) **Linear discriminant analysis** constructs a linear discrimnant function of the input features to best separate the positive class and negative class, assuming the conditional distribution of the input features is Gaussian and the two classes have equal covariance matrices of the input features.

2) **Naïve Bayes** classifier is also a linear classifier. It uses the joint probability of classifying categories and features to estimate the probabilities of categories given feature evidence using Bayes theorem, assuming that the conditional probabilities of the input features are independent given the class label. In our case, we convert every continuous feature such as expression correlation into several binary features by binning the data, and then use these binary features to construct the Naive Bayes classifier.

3) **Linear Support Vector Machine** first maps the inputs features into a high dimension feature space using a linear kernel method. Next to maximize the quality of the output on a training set, Linear SVM identifies an optimal hyperplane dividing the positive and negative labeled samples with the maximum margin of separation. Compared with the above linear classifiers, Linear SVM does not require additional assumptions about the conditional distribution of the input features and represents a more flexible and generalized linear model.

   We use linear SVM to integrate all the six input elements to calculate the FLN linkage weight. First, each of the six individual elements (data sources) is scored as described in the method section of the main manuscript. The FLN linkage weight is the weighted sum of the six elements as described by a linear SVM decision function (equation s1), where x_i denotes the score of data source *i* and *Wi* and *b* are parameters determined by optimizing predictions of pathway sharing for protein pairs in the training set. The detail descriptions for linear SVM are available in Burges et al.'s paper [2].

$$FLN\ linkage\ weight = w_1x_1 + w_2x_2 + w_3x_3 + w_4x_4 + w_5x_5 + w_6x_6 + b \quad (s1)$$

   As a general and flexible linear model, linear SVM does not require the assumption that the input elements are independent. SVM simultaneously adjusts all W_i*s* to optimize the prediction performance over the training data.

4) **Neural network** can be viewed as a simplified model of neural processing in the brain. The nodes in a neural network are organized into weighted directed graphs and divided into multiple layers: input layer, one or more hidden layers, and one output layer. The input layer consists of the input features, each representing one

node. The next layer is called a hidden layer; there may be several hidden layers. The last layer is the output layer, where there is one node for each class (two nodes in our case) corresponding to the positive class and the negative class. Except the input layer, each node receives a signal from all the nodes in the previous layer, sums them up, and multiplies the sum by a specific weight. The resulting signal is then broadcast to all of the nodes in the next layer. A single sweep forward through the network results in the assignment of a value to each output node. The learning process involves updating network architecture and connection weights so that a network can efficiently perform a classification task. Neural Networks represent a complex classification model and can be used to learn non-linear input-output relationships.

2. Training/classification of individual classifiers and classifier aggregation

For each of the 4 machine learning methods we train 30 individual classifiers, each based on a unique segment of the GS set (gold standard set), and followed by aggregation for final predictions.

1) Individual classifiers
   We use an equal number of GSP (gold standard positive) pairs and GSN (gold standard negative) pairs in each training set, Ben-Hur et al. also used balanced GSP and GSN for training in a similar study [3]. Proteins in the GS (gold standard set) are randomly divided into three equal subsets, each contains 386 proteins. For each subset, 4000 GSP pairs and 4000 GSN pairs composed of only proteins in that subset are randomly retrieved from GS set. This serves as a single training set which is composed of 4000 GSP and 4000 GSN. This size is large enough to train a low biased classifier [4] and is also the maximum size allowed (by memory allocation restrictions) in our IBM eServer running the SPIDER machine learning package [5]. In this way we generate 3 individual training sets with non-overlapping proteins, each with 4000 GSN pairs and 4000 GSP pairs. Next, we train a classifier independently on each of the 3 training sets. Each classifier is then applied to all protein pairs in the input set (including both gold standard for validation purpose and non-gold standard pairs). To insure that predictions are only made for out-of-sample pairs, we only retain predicted weights for pairs that were not directly used in the training of the classifier. This leaves us with 3 sets of predicted weights (one from each classifier). To decrease variance in the classifier outputs [4], we repeat the whole process from the beginning 10 times. This generates a total of 30 individual training sets, 30 individual classifiers, and 30 corresponding prediction output sets.

2) Aggregation of individual classifiers for final predictions
   To generate the final predictions, we aggregate the 30 output prediction sets [6]. For each protein pair in the input set we use the median value of the 30 predictions made on that pair. Specifically, the linkage weight of every protein pair (composed of protein A and B) is determined by taking the median of the

output weights of only those left out individual classifiers for which neither protein A nor protein B exists in the relevant training sets. In the final output, every protein pair in the input set, including both non-GS pairs and GS pairs, is assigned a real-valued weight by classifier aggregation. This weight measures functional similarity between the two proteins and is normalized into the range [0, 1]. Next using this final output set, we evaluate the performance of machine learning based data integration.

3. Compare decision rules based on FLNs constructed by the other classifiers

In Figure 5A, we show that the MW decision rule has the highest performance among the four rules based on linear SVM integrated FLN. We further apply the MW rule to FLNs integrated by the other three tested classifiers, and find that this shows consistent results (Fig. S1a-c). This confirms the generality and high performance of the MW decision rule, which is not limited in performance by the type of classifier used to create the FLN.

a

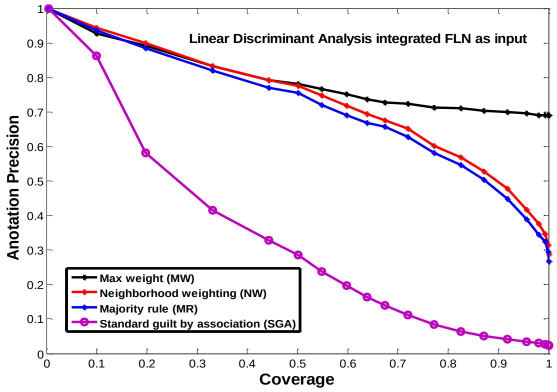

b

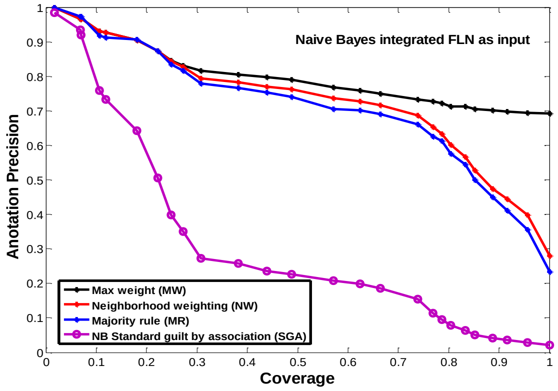

c

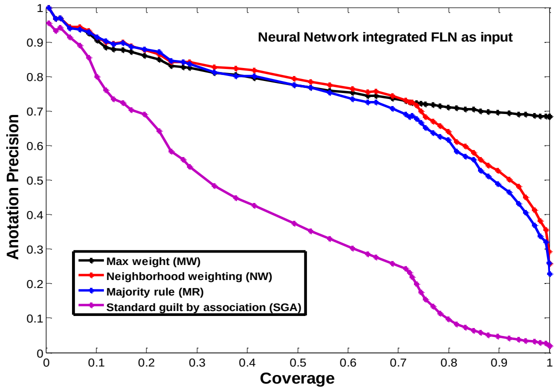

Figure S1: Compare decision rules in FLNs integrated by Linear Discriminant Analysis (a), Naïve Bayes (b), and Neural Network (c). Each x-axis denotes the varied coverage (fraction of proteins having predicted annotations) under varied linkage weight cutoffs. MW has the best performance in all instances.

4. More prediction examples

We assign annotations to proteins that are unannotated in KEGG, though some of these are annotated in other databases such as SGD [7] and MIPs [8]. After MW scores are calibrated into precisions by the fitting function in Fig 9, we illustrate that our predictions with high precisions correspond to correct pathway assignments using the following two novel predictions as examples. More examples are provided in additional file 1.

Example YGL105W demonstrates one protein can have multiple accurate predictions. In the FLN, among all the links connecting YGL105W to annotated neighbors, the links to Mes1p and Gus1p have the first two highest weights of over 0.57. In the KEGG database, as a glutamyl-tRNA synthetase, Gus1p participates in three pathways, “Glutamate metabolism”, “Porphyrin and chlorophyll metabolism”, and “Aminoacyl-tRNA biosynthesis”. As a methionyl tRNA synthetase, Mes1p also participates in three pathways, “Methionine metabolism”, “Selenoamino acid metabolism”, and “Aminoacyl-tRNA biosynthesis. The number of union pathways of Gus1p and Mes1p is five since they both participate in aminoacyl-tRNA biosynthesis. The MW decision rule assigns

these five pathways to YGL105W as the top five ranked predictions, all with a high precision of over 0.7. In SGD, YGL105W is described as "Protein that binds tRNA and methionyl-synthetases and glutamyl-tRNA synthetases(Mes1p and Gus1p), delivering tRNA to them, stimulating catalysis, and ensuring their localization to the cytoplasm". This suggests that YGL105W does participate in those five pathways by assisting Gus1p and *MES1p*.

Our method can predict functions for uncharacterized proteins such as YDR539W (an uncharacterized protein in SGD). In the FLN, among the links connecting target YDR539W to annotated neighbors, the link to Pad1p (encodes *UbiX* protein, a 3-octaprenyl-4-hydroxybenzoate carboxy-lyase in ubiquitone biosynthesis) has the highest linkage weight of 0.75 due to their highly correlated phylogenetic profiles. MW assigns the pathway of Pad1p, "Ubiquinone biosynthesis", as the top ranked prediction with a high precision of 0.97. All other predictions have a low precision of less than 0.05. We also find additional evidence supporting this prediction as listed below. First, Pfam [9] detects a single *UbiD* domain (E-value 2e-109) in YDR539Wp. Second, YDR539W and *PAD1* are located adjacently in chromosome IV with 464 bases apart. Third, based on EcoCyc [10], *E. coli* has two 3-octaprenyl-4-hydroxybenzoate carboxy-lyases in ubiquitone biosynthesis, including *E. coli UbiX* and *E. coli UbiD* , which are indicated to function in different physiological conditions [11]. YDR539Wp and Pad1p are homologous to *E. coli UbiD* protein and *UbiX* protein, respectively (blast E-value 1e-31 and 5e-45, respectively), while there are no sequence similarity between *E. coli UbiD* and *E. coli UbiX*, or between YDR539Wp and Pad1p. All these additional evidence supports that YDR539W participates in "ubiquitone biosynthesis" pathway as a 3-octaprenyl-4-hydroxybenzoate carboxy-lyase and probably functions in different cellular conditions from the other carboxy-lyase Pad1p. Although by BLAST search YDR539Wp is shown to be homologous to the *UbiD* protein in other organisms, our prediction provides strong additional evidence.

5. Compare the four decision rules for functional annotation in *E. coli*

To further test the generality of the MW decision rule in *E. coli* we also compare the performance of MW and the other three decision rules by integrating six data sources using linear SVM. The six data sources are described below. The training/classification/validation process for machine learning integration is performed similarly as in yeast.

5.1 Data sources in E. coli

Except sequence similarity and expression data, all the pair-wise interaction data are downloaded from Prolink database with the P-values as input features [12].

1) Sequence similarity
   In a similar procedure as yeast, we generate 8172 similar pairs involving 2494 proeins in *E. coli*.

2) Protein domain fusion

Totally 8178 fusion pairs involving among 1166 proteins are included.

3) Phylogenic profile
   Total 9475 pairs in 1657 proteins are included.

4) Gene cluster
   In bacteria, genes with closely related functions are often transcribed from the same operon and these genes are located near each other on the same DNA strand. The gene cluster method is applied to detect such pairs [13]. Total 2787 pairs among 3593 genes are incorporated.

5) Gene neighbor data
   Some operons within a particular organism are conserved across other organisms and this conservation can infer functional similarity [12]. Totally 13,916 pairs in 3159 genes are included.

6) Expression data are composed of composed of 4345 genes in 189 experimental conditions [14]. The Pearson correlation coefficient for every pair is computed as the input feature.

When selecting protein pairs used for inputs, similar as in yeast we only include proteins that have both protein sequences available from RefSeq and an expression profile measure. We cover 8,448,105 pairs among 4111 proteins in total. We use 1366 proteins among 110 pathways in *E. coli* as annotation sources (downloaded from KEGG in March, 2007 [15]).

5.2 Gold standard (GS) for machine learning classifiers in E. coli

We define our true negatives gold standard (GSN) as the collection of pairs that: (1) are annotated in KEGG; (2) never occur in the same KEGG pathway based on current knowledge. For prokaryotic *E. coli*, there are no organelles and thus no further filtering GSN by co-localization. *E. coli* GS are composed of 21,899 TPs and 477,601 TNs.

5.3 Results

Similarly as in yeast, in *E. coli* we compare the four decision rules using linear SVM integrated FLN as input and MW also shows superior performance with high annotation precision and high coverage (Fig. S2).

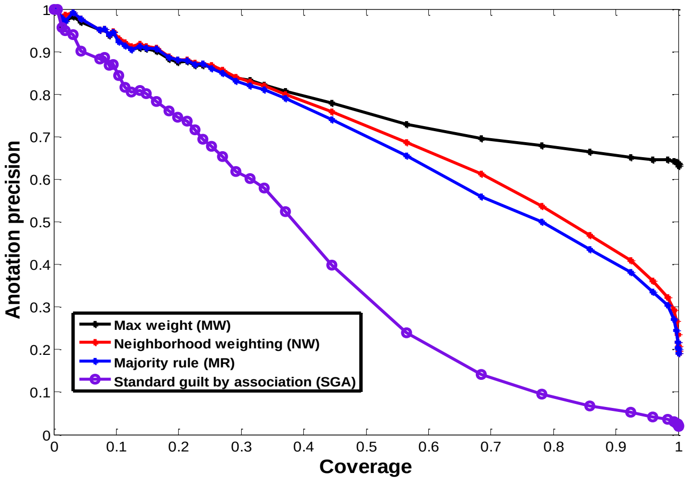

Figure S2: Compare performances of MW, NW, MR, and SGA in *E. coli*, with Linear-SVM integrated FLN as input. The x-axis denotes the varied coverage (fraction of proteins having predicted annotations) under varied linkage weight cutoffs. The y-axis denotes annotation precision. MW shows superior performance to other decision rules.

6. Compare decision rules in annotation precision-recall analysis

6.1 Procedures to plot the precision-recall curve

Precision-recall analysis is commonly used for performance evaluation. Since MW, NW, MR, and functional flow method [16] all provide a weighted annotation score for each prediction (equation 1 and 2), we have plotted the precision-recall curves by thresholding the annotation scores to compare decision rules. The precision and recall are defined in equation 4a and 4b in the manuscript.

As described in the manuscript, linkage weight cutoff and prediction rank both affect annotation performance (Fig. 4). Therefore we take these two factors into account when plotting precision-recall curves.

First we apply a linkage weight cutoff to generate a filtered FLN. Two representative linkage weight cutoffs were chosen, such that we could assess the performance of the decision rules when applied to (i) a dense and high-coverage network and (ii) a sparse and low-coverage network, having low and high linkage weight cutoffs respectively. Second, in leave one out setting (described in page 24, main manuscript), application of a given decision rule to a filtered FLN generates a prediction list for each protein. This list is subsequently sorted in descending order with respect to the annotation scores associated with each method (Equation 1, Equation2, and Fig. 1B). Third, since a protein can have multiple annotations, an arbitrary top 5 rank cutoff is applied, such that at most 5 predictions for each protein are considered in the prediction pool for the precision-recall curve. Schwikowski, et al. applied a similar procedure for selecting predictions with a fixed rank cutoff [17]. Last, a precision-recall curve is plotted for each decision rule by thresholding the respective annotation scores. As result, under two representative linkage weight cutoffs, the precision recall curves are evaluated in two representative

FLNs. One covers 5475 proteins (low cutoff) and the other covers 1872 proteins (high cutoff). Since the linkage weight is proportional to linkage precision (Fig. 3), the first FLN represents a high-coverage and relatively noisy network and the second FLN represents a low-coverage and relatively high-precision network.

6.2 Results using GO slims as annotation ontology

We also have done similar evaluations using GO slim terms as the annotation ontology. Specifically we choose the biological process subcategory (32 terms covering 3815 proteins, downloaded from SGD) since GO biological process is most similar to KEGG pathways. The precision-recall curves showed similar trends as using KEGG (Fig. 5B and Fig. 5C).

a **b**

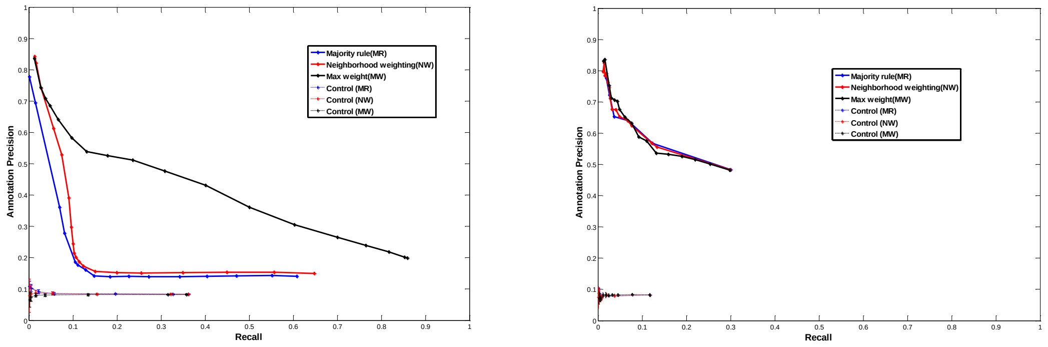

Figure S3: Compare performances of MW, NW, and MR in annotation precision-recall analysis using (**a**) At a high-coverage and relatively noisy FLN with GO slim (biological process) as annotation ontology. (**b**) At a low-coverage and relatively high-precision FLN with GO slim (biological process) as annotation ontology. In all the figures, the same evaluations are repeated in random control networks as dashed lines with error bars indicating one standard deviation.

7. Compare MW decision rule and functional flow algorithm

7.1 Description of functional flow algorithm

The functional flow algorithm simulates function annotation as functional flow between proteins and handles one function at a time. For each function, this algorithm treats each protein annotated with the function as the source of a functional flow and assigned it with an infinite potential. All the other proteins are assigned with 0 potential. Functional flows are then iteratively spread in the network from proteins with higher potential to their immediate neighbors with lower potential. The amount of flow depends on the

linkage weight of the respective edge. The number of iteration for function-flow spread is represented by a parameter "d ", which determines the maximum propagation distance from a source node to a recipient node. The annotation score of an unannotated protein is the total amount of flow it receives from all the immediate neighbors during the d iterations. When d is set to be 1, the function predictions for a target protein are derived only from the functional links to its immediate neighbors with known functions. When d is set to be 2 or above, the proteins with function predictions in (d -1) iteration can serve as additional annotation sources for function propagation. We implement the functional flow algorithm according to the detailed descriptions of the original paper [16].

7.2 Comparison results

We compare MW and functional flow method using annotation precision-recall curves as described in page 7-8 of the supplemental material. The results are as below.

(i) At a low linkage-weight cutoff (a high-coverage and relatively noisy network), MW outperforms functional flow, the precision difference growing to over 0.4 at maximum recall of the functional flow method (Fig. S4a, supplemental material). (ii) At a high linkage-weight cutoff (low-coverage and relatively confident network) both methods have high annotation precision (above 0.8) and low recall (below 0.42) (Fig. S4b, supplemental material). In both conditions, increasing d does not improve the performance for functional flow method. For the purpose of providing precise annotations covering as many proteins as possible, MW is preferable.

Functional flow method uses global network connectivity for step by step functional propagation, at each step it uses a local decision rule to increase the annotation score for a target protein using the sum of flows from its immediate neighbors with a higher annotation-source reservoir.

We believe that the poor performance of functional flow method at high coverage can be explained by the local decision rule, underlying the global scheme. A direct comparison of MW with the local rule underlying functional flow can be done by setting the d parameter to 1, which corresponds to limiting propagation of information to adjacent nodes, and in effect making functional flow a local method. As shown in Fig. S4a, the functional flow algorithm (d =1) is substantially outperformed by the MW rule. Therefore, although functional flow method incorporates more information in each annotation by means of global propagation, its inappropriate local rule limits its performance in our FLN. We feel that the comparison of functional flow and MW illustrates the important point that despite the potential benefits of global annotation schemes, dedicated studies of local decision rules are still of great importance, as local rules are critical in determining performance of global methods. In the future, we plan to incorporate the MW rule into a global propagation scheme and we expect the performance will likely improve further.

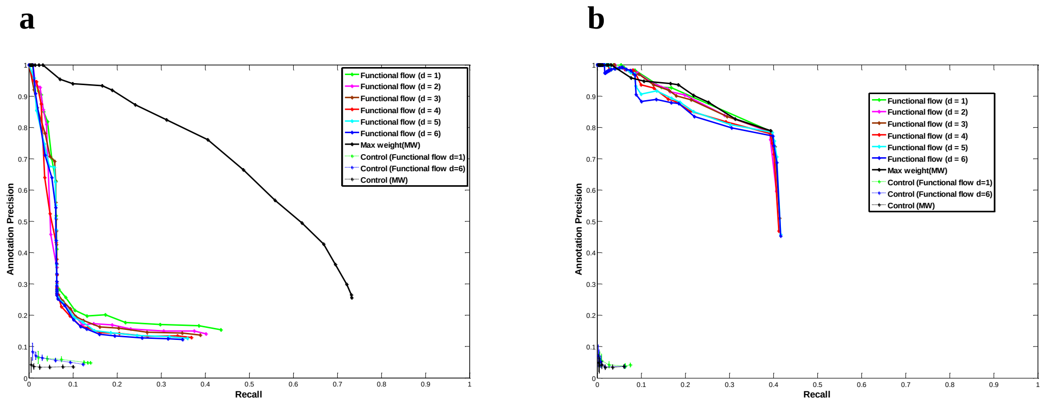

Figure S4: Compare performances of MW and functional flow method in annotation precision-recall analysis. For functional flow method, d represents the maximum propagation distance from a source node to a recipient node as described in the original paper. (**a**) At a high-coverage and relatively noisy FLN. (**b**) At a low-coverage and relatively high-precision FLN. In all the figures, the same evaluations are repeated in random control networks as dashed lines with error bars indicating one standard deviation.

8. Test robustness of the framework by reducing data sources or annotation sources

In order to more rigorously assess whether our framework is likely to meet with success when applied to organisms where less data and annotation are available we performed the following analyses in yeast and *E. coli*:

(1) First we test the robustness of MW method against decreased annotated (seeded) nodes given the same functional linkage network (FLN). Instead of using all the KEGG proteins (1161 KEGG proteins for yeast and 1366 KEGG proteins for *E. coli*), we randomly choose 500, 200, and 100 KEGG proteins as annotation source and apply the MW decision rule for functional annotations, each with 10 repeating runs and the final results are determined by the median performance. We find the precision at maximum coverage drops from the original 0.71 to 0.58, 0.46 and 0.38 in yeast and from the original 0.63 to 0.48, 0.36, and 0.27, in *E. coli*. (Fig S5a and S5b).

(2) Since the vast majority of prokaryotic organisms do not have expression data sets available and most eukaryotes don't have high-throughput PPI (protein-protein physical interaction) or GI (genetic interaction) data available, we want to see how performance is affected by leaving out expression data in *E. coli* and leaving

out PPI and GI data in yeast. Specifically, in *E. coli* we only use sequence based annotation sources (e.g. phylogenetic profiling, protein domain fusion, chromosomal proximity, gene cluster, sequence similarity, page 5-6 of supplemental material), which are readily available for any sequenced prokaryotic organism. In yeast we use only expression, sequence similarity, phylogenetic profile, and protein domain fusion as eukaryotes usually have both sequence data and expression data available. We find in *E. coli* the maximum coverage drops from full coverage to 0.75, but the annotation precision remains over 0.7 at various degrees of coverage (Fig. S5d). For yeast the precision drops from 0.71 to 0.6 at maximum coverage and remains over 0.6 at various degrees of coverage (Fig. S5c).

In both tests the dropped performances are small relative to the overall performances, and it is therefore likely that our framework can be applied to a less studied organism.

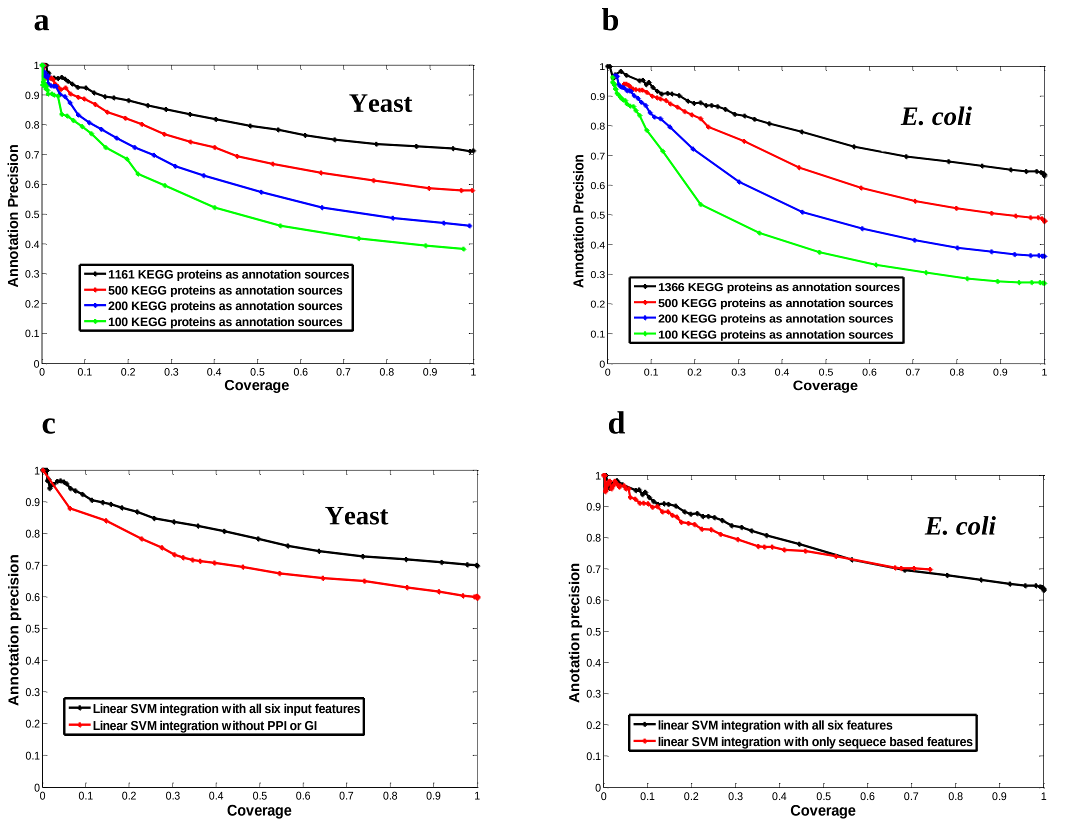

Figure S5: Test robustness of the framework by reducing data sources or annotation sources using annotation precision-coverage curves. Randomly choose 500, 200, and 100 annotated KEGG proteins as annotation sources in yeast (**a**) and *E. coli* (**b**) and compare the performance as use all the KEGG proteins. (**c**) In yeast remove PPI (protein-protein physical interaction) and GI (genetic interaction) data from the input data sources and compare the performance as use all the data sources. (**d**) In *E. coli*, remove expression

data and use only sequence based data sources to compare the performance as use all the data sources.

In Figure S5a, the annotation precision at max coverage in yeast is around 0.4 with just 100 annotated nodes. The interpretation for this relatively high performance is discussed as follows.

In the above tests, we randomly select the seeded nodes from the 1161 KEGG proteins with the assumption that each KEGG protein has equal probability to be selected as seed. Therefore, 100 selected seeds still cover most of KEGG pathways (67 out of 99). In a real situation however, if only 100 proteins are annotated in an organism, it is likely that these proteins will not be randomly distributed across all categories of KEGG pathways. Specifically, one might expect that these proteins would cluster in a few well understood pathways, thereby reducing the diversity of the seed proteins. Such a decrease in seed diversity would certainly result in a decrease in both the precision and coverage relative to our estimated values.

We also want to point out that the annotation precision-coverage curve was plotted with the assumption that all the 5475 proteins in the functional linkage network could be assigned to at least one of the 99 existing KEGG pathways. Similar precision versus coverage evaluations were also used to estimate functional annotation performance or functional linkage inference by other research groups [18-21]. However, since current KEGG pathway database is far from complete and is under continuous expansion, it is likely that only a subset of the unannotated proteins within the 5475 proteins can be assigned to an existing KEGG pathway. For those other proteins not belonging to any existing KEGG pathway, the annotation precision for them would be 0. As a result, the actual precision at max coverage should be lower than our estimation. Unfortunately, it is not possible to estimate this actual precision, because we do not know what fraction of the proteins encoded in a genome belongs to existing KEGG pathways and what fraction does not.

In summary, we do not claim that our method could definitely reach a precision of around 0.4 at genome coverage with just 100 annotated seeds. Instead, the precision should be understood as an upper bound estimation considering the above assumptions. With the continuous growth of KEGG database and more available KEGG proteins and KEGG pathways, we would be able to estimate the KEGG-pathway predictions more accurately in the future.

9. Perform random control experiments for each evaluated annotation method.

We have added corresponding random control curves for all the methods evaluated in Figure 5 of the main manuscript, Figure S3 and S4 of the supplemental material.

To generate the random control networks, after the construction of a functional linkage network by data integration, we randomize the edges such that the new weights have nothing to do with the original proteins they connected. Next, we compare different

annotation methods using the same evaluation procedures as before in the random control network. The whole process is repeated 10 times and 10 random networks are generated. The final random control curve for each method is determined by the mean performance in the 10 random control networks with error bars indicating one standard deviation.

The results are summarized as below. For each annotation method, annotation precision is markedly lower when using a random control network (precision is less than 0.15 at all tested thresholds) when compared with the results from the real network (precision can be over 0.9 at stringent thresholds). In addition, for each annotation method the maximal recall of the random network is also lower than that of the real network. This decrease in recall can be explained as follows. For each protein we used only its top 5 ranked annotations for evaluation (see annotation precision recall curve description on page 7 of supplemental material). Since the top 5 annotations in the random control network have much lower true positive rates than real network, the recall for the random network is lower than real network according to Equation 4b. In summary, relative to performances with the real network, the random control curves for different annotation methods have roughly similar poor performances. MW random control does NOT have a higher performance than other methods. Therefore, we conclude that our evaluations do NOT bias towards MW.

Reference

1. Richard O. Duda, Peter E. Hart, Stork aDG: **Pattern Classification**, 2nd edn. New York, NY: Wiley-Interscience; 2000.
2. Burges CJC: **A Tutorial on Support Vector Machines for Pattern Recognition**. *Data Mining and Knowledge Discovery* 1998, **2**:121 - 167.
3. Ben-Hur A, Noble WS: **Choosing negative examples for the prediction of protein-protein interactions**. *BMC Bioinformatics* 2006, **7 Suppl 1**:S2.
4. Kohavi R: **A study of cross-validation and bootstrap for accuracy estimation and model selection**. *Proceedings of the Fourteenth International Joint Conference on Artificial Intelligence* 1995, **2**(12):1137-1143.
5. **The Spider: http://www.kyb.tuebingen.mpg.de/bs/people/spider/**.
6. Barutcuoglu Z, Schapire RE, Troyanskaya OG: **Hierarchical multi-label prediction of gene function**. *Bioinformatics* 2006, **22**(7):830-836.
7. **The Saccharomyces Genome Database: http://www.yeastgenome.org/**.
8. **Munich information center for protein sequences: http://mips.gsf.de/**.
9. Finn RD, Mistry J, Schuster-Bockler B, Griffiths-Jones S, Hollich V, Lassmann T, Moxon S, Marshall M, Khanna A, Durbin R *et al*: **Pfam: clans, web tools and services**. *Nucleic Acids Res* 2006, **34**(Database issue):D247-251.
10. **Encyclopedia of Escherichia coli K -12 Genes and Metabolism: http://ecocyc.org/**.
11. Zhang H, Javor GT: **Regulation of the isofunctional genes ubiD and ubiX of the ubiquinone biosynthetic pathway of Escherichia coli**. *FEMS Microbiol Lett* 2003, **223**(1):67-72.

12. Bowers PM, Pellegrini M, Thompson MJ, Fierro J, Yeates TO, Eisenberg D: **Prolinks: a database of protein functional linkages derived from coevolution**. *Genome Biol* 2004, **5**(5):R35.
13. Overbeek R, Fonstein M, D'Souza M, Pusch GD, Maltsev N: **The use of gene clusters to infer functional coupling**. *Proc Natl Acad Sci U S A* 1999, **96**(6):2896-2901.
14. Faith JJ, Hayete B, Thaden JT, Mogno I, Wierzbowski J, Cottarel G, Kasif S, Collins JJ, Gardner TS: **Large-scale mapping and validation of Escherichia coli transcriptional regulation from a compendium of expression profiles**. *PLoS Biol* 2007, **5**(1):e8.
15. **Kyoto encyclopedia of Genes and Genomes: http://www.genome.jp/kegg/**.
16. Nabieva E, Jim K, Agarwal A, Chazelle B, Singh M: **Whole-proteome prediction of protein function via graph-theoretic analysis of interaction maps**. *Bioinformatics* 2005, **21 Suppl 1**:i302-310.
17. Schwikowski B, Uetz P, Fields S: **A network of protein-protein interactions in yeast**. *Nat Biotechnol* 2000, **18**(12):1257-1261.
18. von Mering C, Huynen M, Jaeggi D, Schmidt S, Bork P, Snel B: **STRING: a database of predicted functional associations between proteins**. *Nucleic Acids Res* 2003, **31**(1):258-261.
19. Lee I, Date SV, Adai AT, Marcotte EM: **A probabilistic functional network of yeast genes**. *Science* 2004, **306**(5701):1555-1558.
20. McDermott J, Bumgarner R, Samudrala R: **Functional annotation from predicted protein interaction networks**. *Bioinformatics* 2005, **21**(15):3217-3226.
21. Wu J, Hu Z, DeLisi C: **Gene annotation and network inference by phylogenetic profiling**. *BMC Bioinformatics* 2006, **7**:80.
